# Supplementary material for: New seasonal pattern of pollution emerges from changing North American wildfires
Source: Nat Commun. 2022 Apr 19;13:2043. doi: 10.1038/s41467-022-29623-8 (PMC9018720; doi:10.1038/s41467-022-29623-8)
Supplement: Supplementary file 1 — Supplementary Information [file 41467_2022_29623_MOESM1_ESM.pdf]

Supplementary information for:
New seasonal pattern of pollution emerges from changing North American wildfires

Rebecca R. Buchholz[1], Mijeong Park[1], Helen M. Worden[1], Wenfu Tang[1], David P. Edwards[1], Benjamin Gaubert[1], Merritt Deeter[1], Thomas Sullivan[2], Muye Ru[3], Mian Chin[4], Robert C. Levy[4], Bo Zheng[5] & Sheryl Magzamen[6]

1. Atmospheric Chemistry Observations & Modeling Laboratory, National Center for Atmospheric Research, Boulder, CO, USA
2. University of Colorado, Boulder, CO, USA
3. Columbia University, New York, USA
4. NASA Goddard Space Flight Center, Greenbelt, MD, USA
5. Institute of Environment and Ecology, Tsinghua Shenzhen International Graduate School, Tsinghua University, Shenzhen 518055, China
6. Colorado State University, Fort Collins, CO, USA

Contents

1 Supplementary Figures **2**

2 Supplementary Information Section 1: Global model simulations **8**

3 Supplementary Information Section 2: Potential health relationships **13**

1 Supplementary Figures

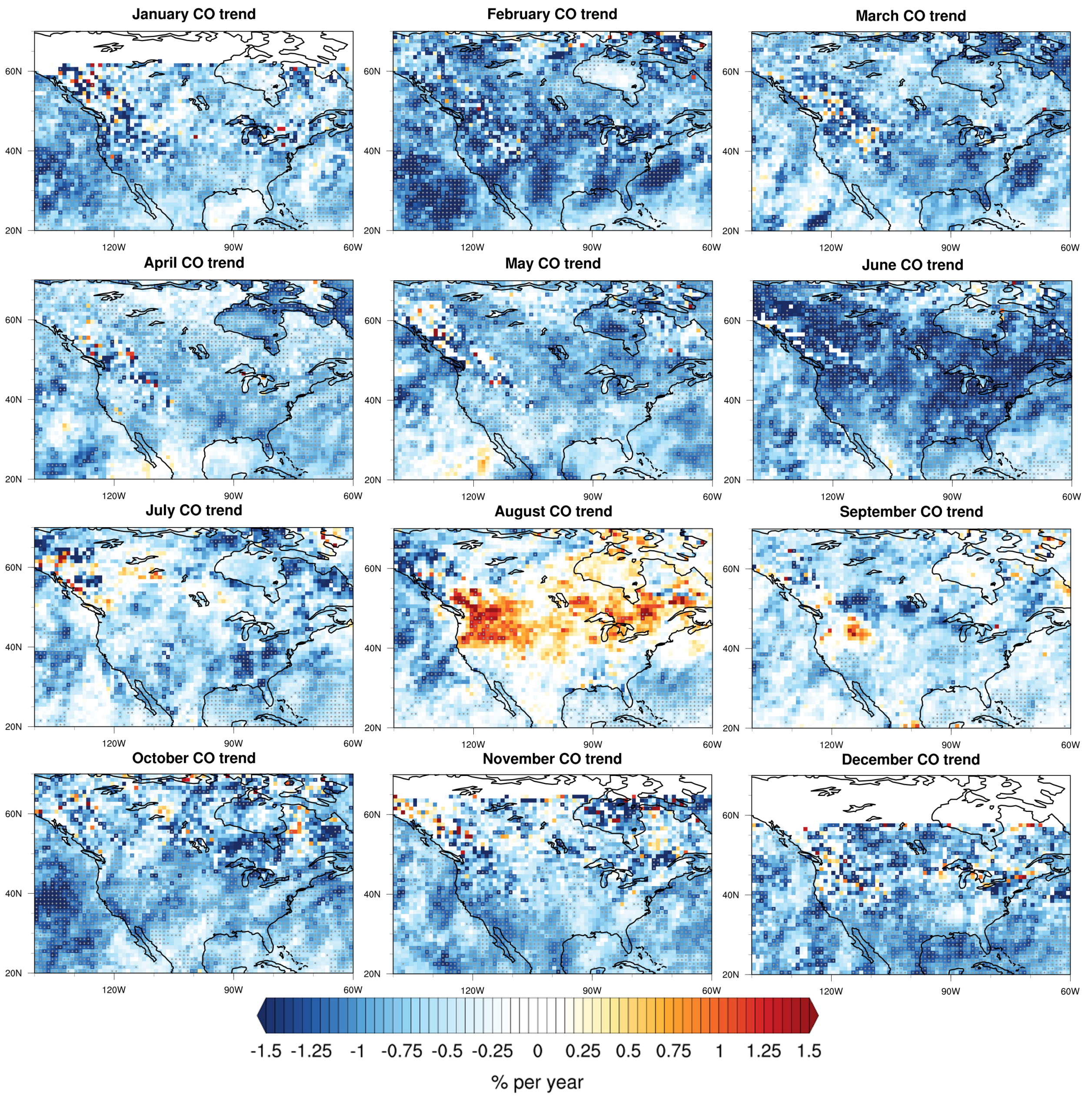

Supplementary Figure 1: Maps of trend in column CO between 2002-2018 by month. Grey dots indicate a probability of nonzero trends to 95%

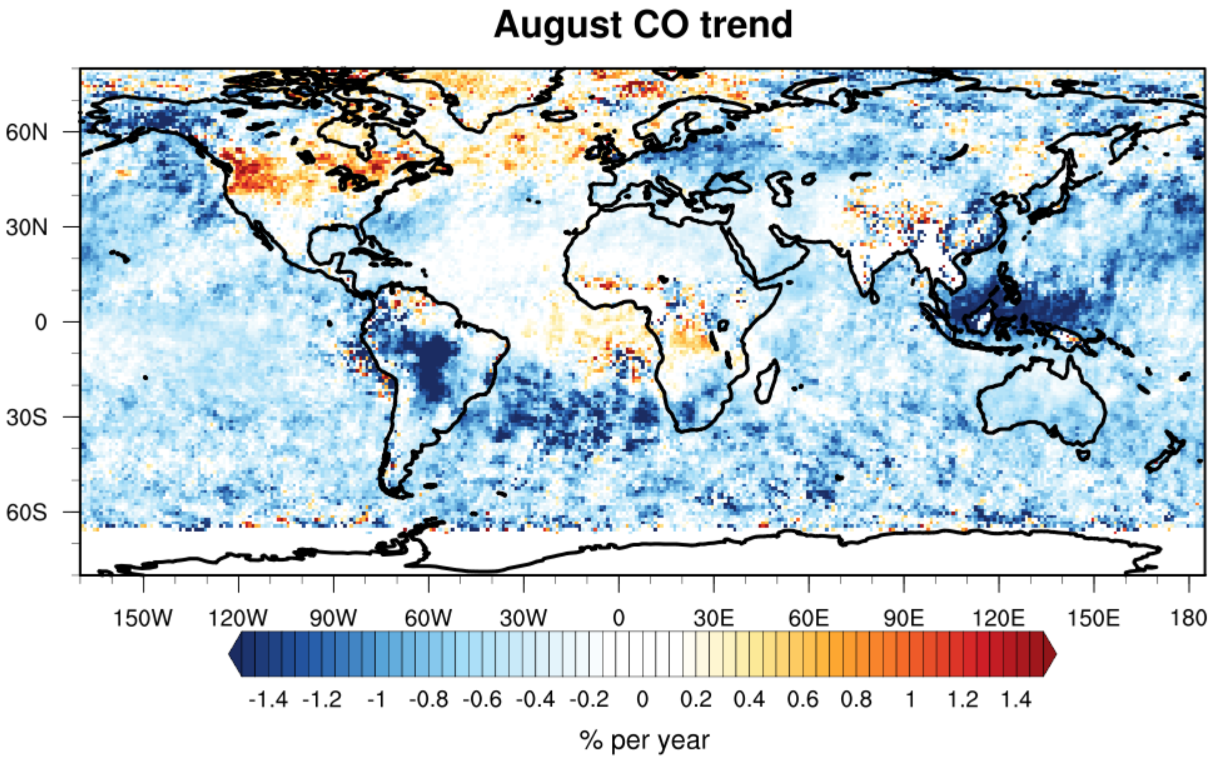

Supplementary Figure 2: Global map of trend in August column CO between 2002-2018.

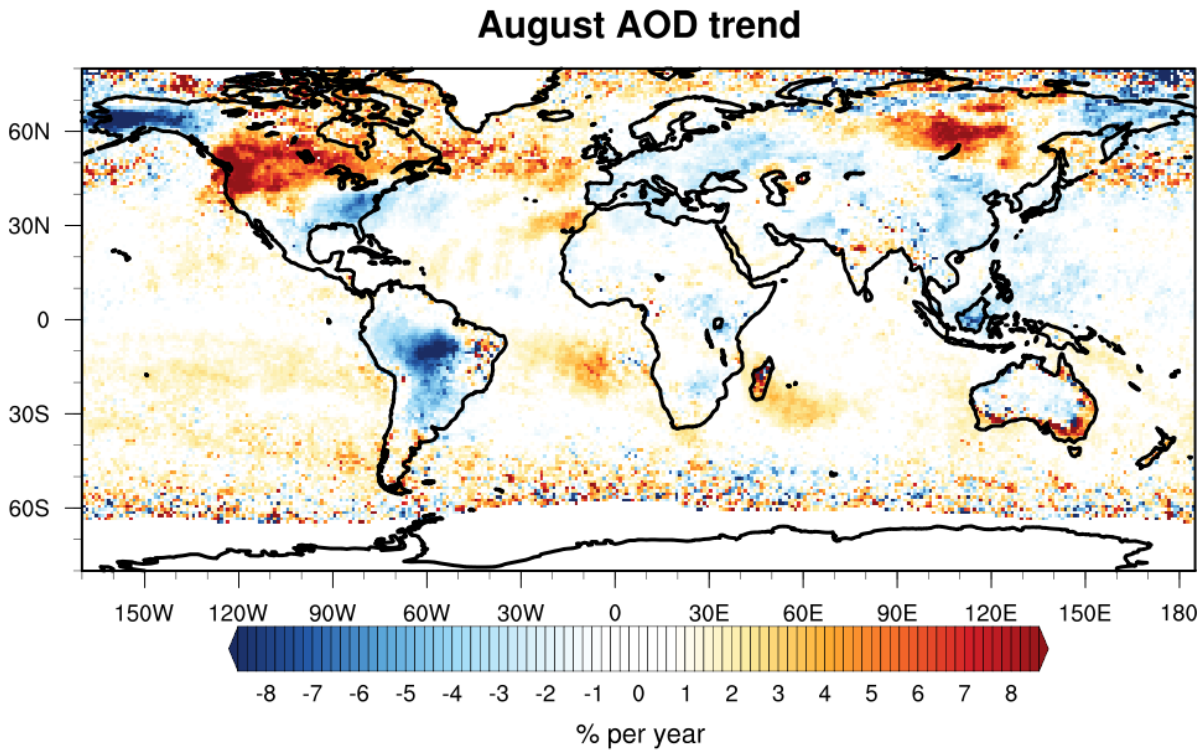

Supplementary Figure 3: Global map of trend in August AOD between 2002-2018.

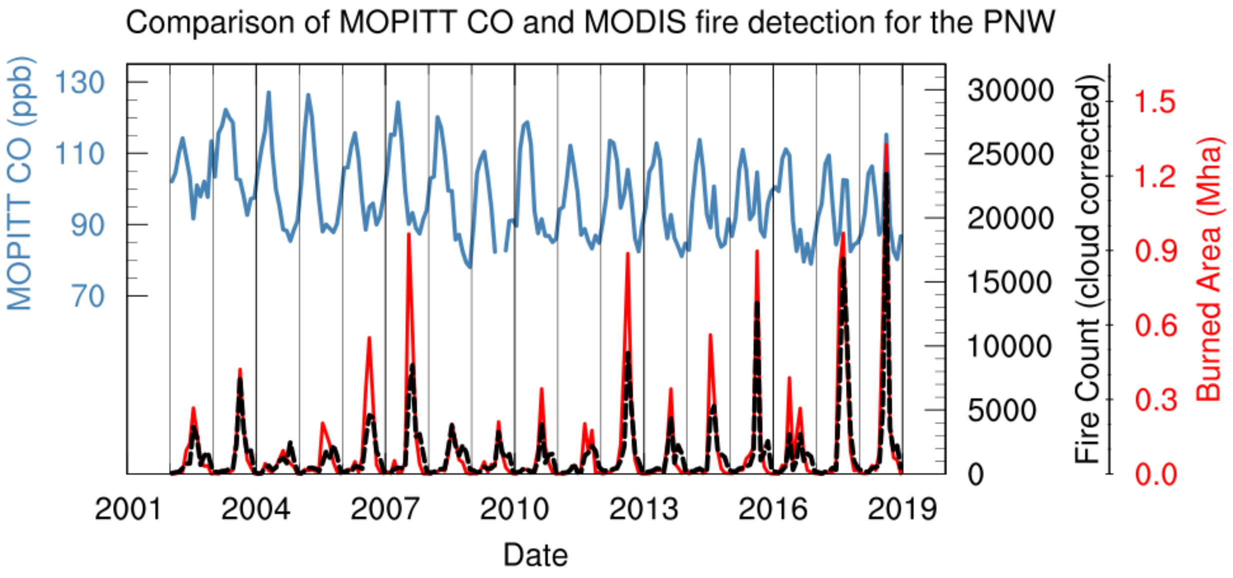

Supplementary Figure 4: Comparison of MOPITT CO and MODIS fire detection for the PNW. Time series of PNW monthly average MOPITT CO (blue) and monthly summed MODIS fire detection for burned area (red, MCD64CMQ[1]) and fire count (black, MOD14CMQ[2]) in the same region. Vertical lines show the beginning of each year.

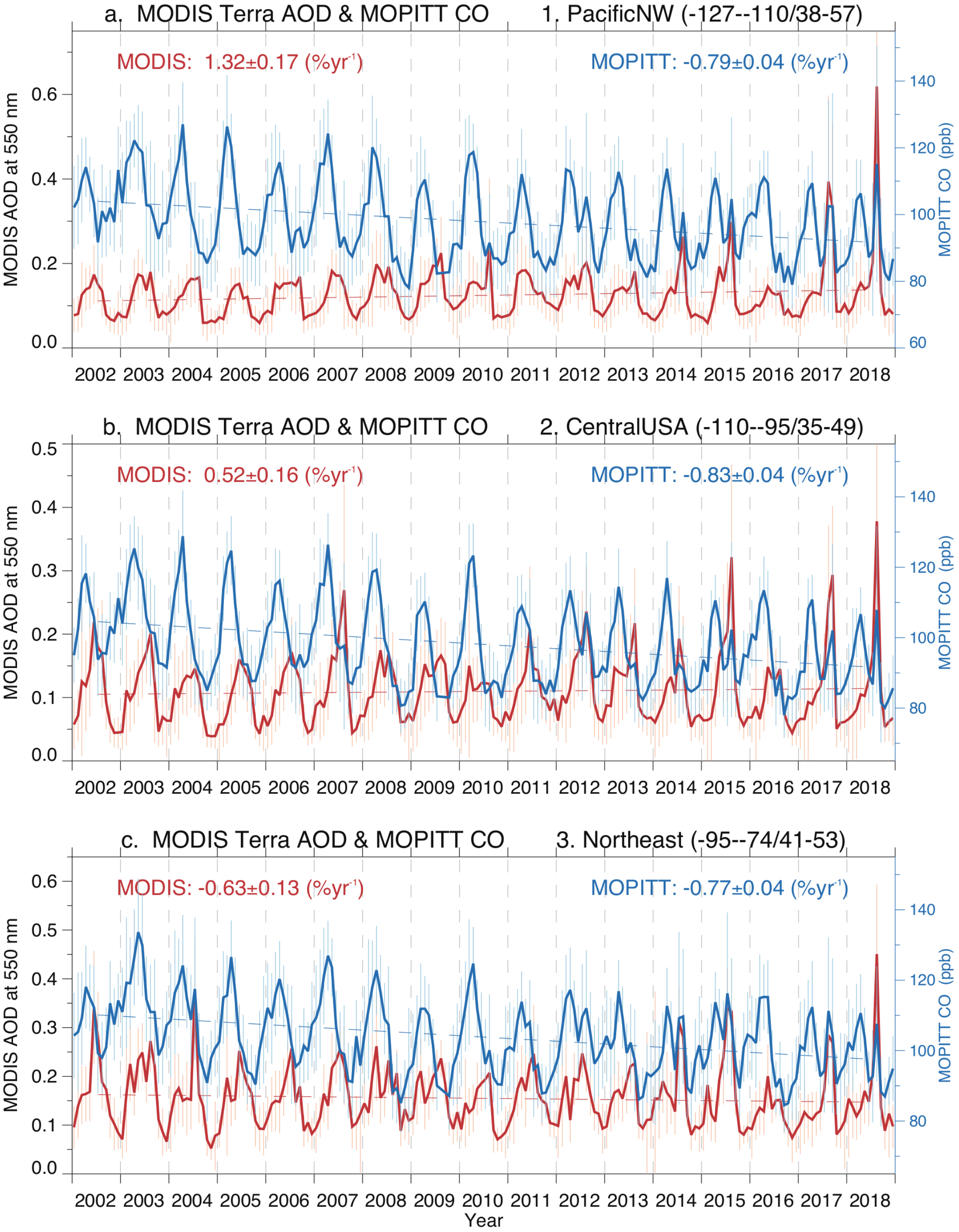

Supplementary Figure 5: Time series and trend analysis of CO and AOD in studied regions. For the CO observations, which are regional averages of all retrievals within a month, the mean sample size is PNW: 1110, Central USA: 2469, and Northeast: 1341. For the AOD data, which are regional averages of monthly average data, the mean sample size is: PNW: 240, Central USA: 190 and Northeast: 175.

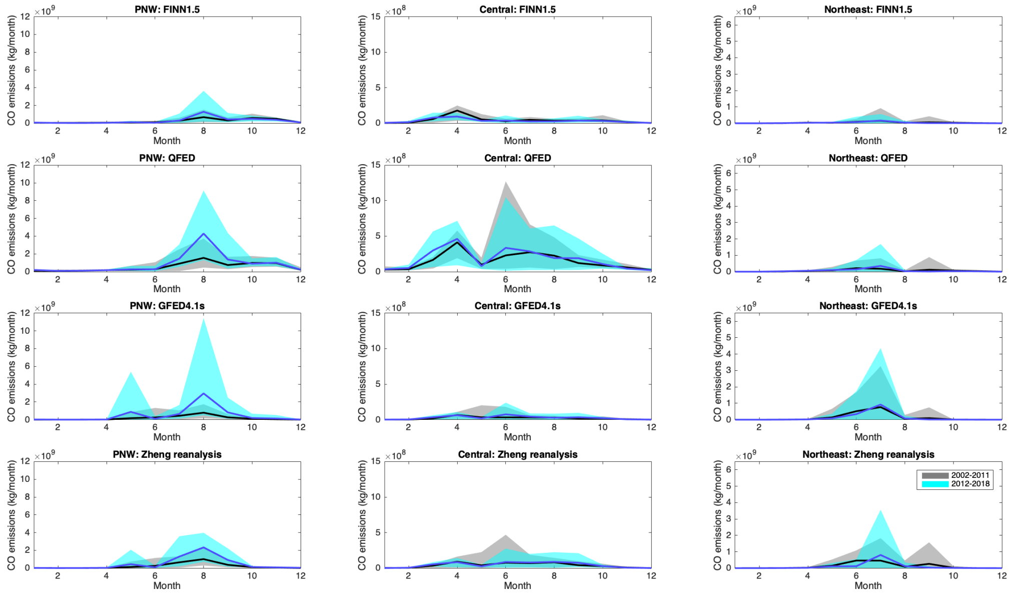

Supplementary Figure 6: Seasonal cycle analysis of wildfire emissions. Seasonal cycles of wildfire emissions of CO for the PNW (left), Central USA (center), and the Northeast (right) regions based on FINN1.5[3], QFED[4, 5], GFED4.1s[6, 7], and Zheng reanalysis[8]. Black lines are averaged monthly mean for 2002-2011. Blue lines are averaged monthly mean for 2012-2018 where data is available (Zheng ends in 2017). Shading represents the range in inter-annual variability for each month.

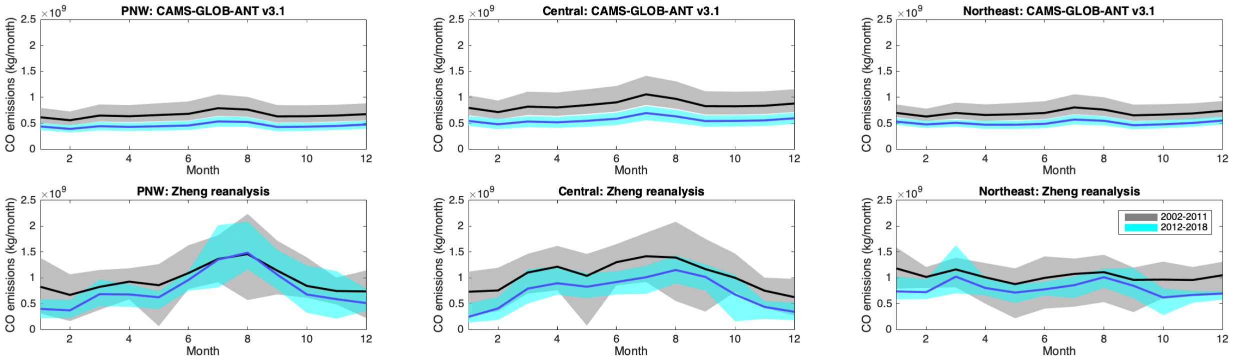

Supplementary Figure 7: Seasonal cycle analysis of anthropogenic emissions. Same as Supplementary Figure 6 but for anthropogenic emission inventories CAMS-GLOB-ANT v3.1 and Zheng reanalysis[8]. Anthropogenic emissions of CO from Zheng et al. (2019)[8] are based on multi-species atmospheric inversions, using CEDS[9] as prior information for anthropogenic emissions and constrained by satellite retrievals of atmospheric compositions (e.g., MOPITT CO).

2 Supplementary Information Section 1: Global model simulations

We use global simulations with the Community Earth System Model version 2 (CESM2) implementing the Community Atmosphere Model with chemistry (CAM-chem) to support this study in two ways. First, we look at a long time period of simulation and compare emissions and burden to investigate the impact of PNW changes on other regions. Second, we directly explore the potential for PNW wildfire emissions to impact surface air over North America.

The CAM-chem simulations over a long time period (2002-2018) uses QFED fire emissions with the setup as described in Emmons et al. (2020)[10]. Subtracting the mean surface CO of the early period (2002-2011) from the later period (2012-2018) shows large enhancements over the PNW and extending across North America (Supp. Fig. 8). Hydrogen cyanide (HCN) is predominantly emitted from fires and is also simulated by CAM-chem. The similar pattern of HCN to CO in the equivalent difference plot (Supp. Fig. 9) supports that the increase in the PNW CO for the later period is driven by wildfires.

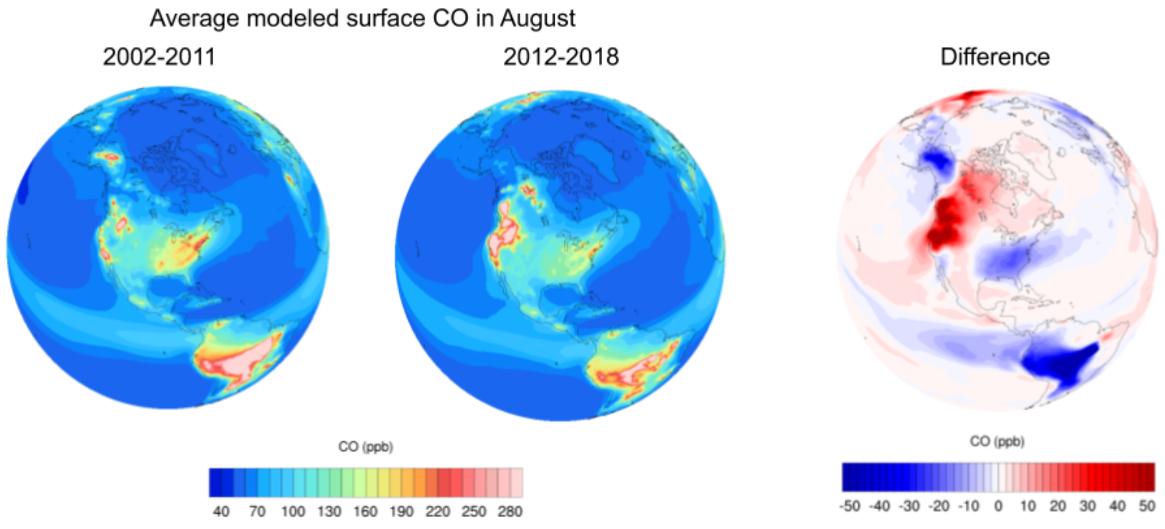

Supplementary Figure 8: Analysis of modeled CO. Average August CAM-chem simulations of surface layer CO from 2002-2011 (left), 2012-2018 (center), and their difference (right).

The burden of CO over the whole column is calculated for the 3 regions of the main paper, as well as for an inflow region over the Pacific, which has the same volume as the PNW (Supp. Fig. 10). Burden calculations include emissions, deposition, chemical production and loss,

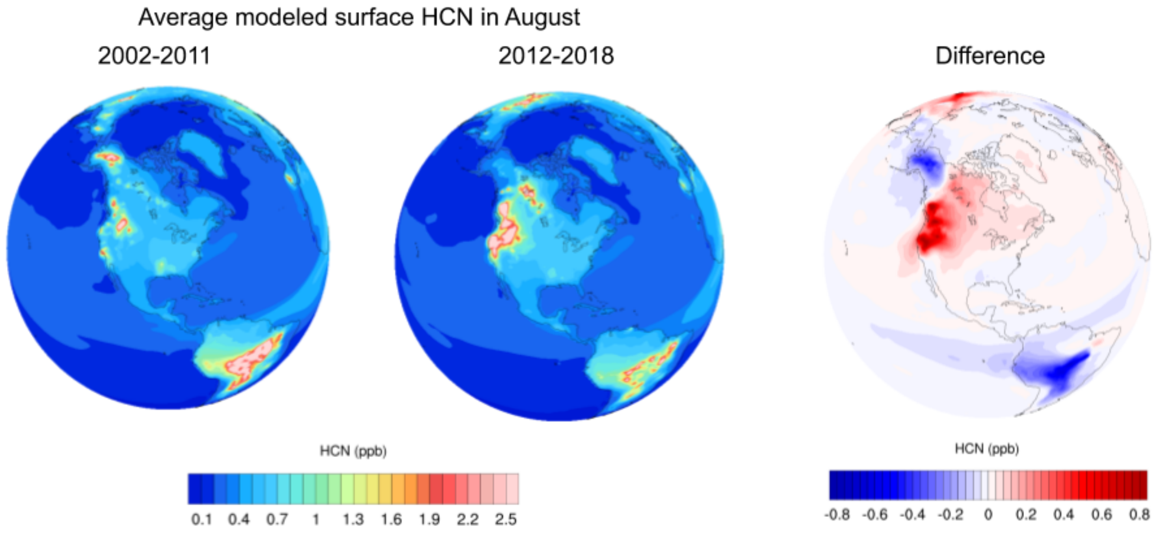

Supplementary Figure 9: Analysis of modeled HCN. Average August CAM-chem simulations of surface layer HCN from 2002-2011 (left), 2012-2018 (center), and their difference (right).

to provide the total mass inside a region.

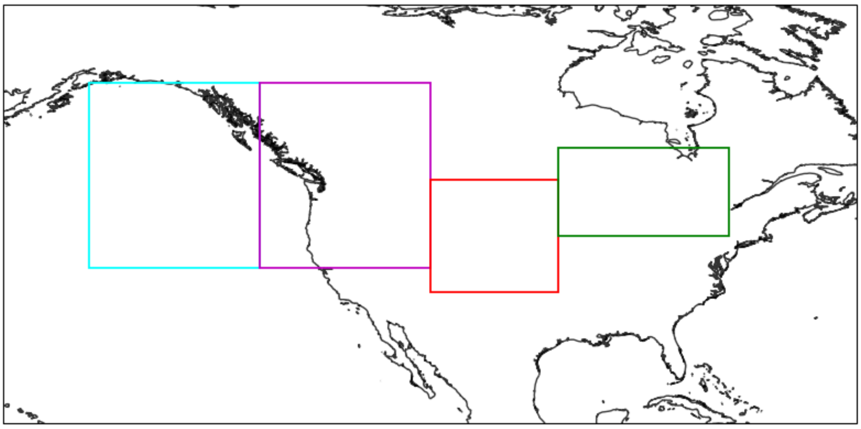

Supplementary Figure 10: Domains used for analysis of CAM-chem simulations. The Pacific region (light blue) is in addition to the regions used in the main manuscript.

Emissions and burden for the August CO regional averages for 2002-2011 compared to 2012-2018 are shown in Supplementary Figure 11. Over the PNW, the signal from the strong increase in emissions is seen through an increase in the burden. No difference in the incoming

CO burden (Eastern Pacific) further points to an increase in emissions over the PNW driving the increase in the PNW burden. For the Central USA and Northeast regions, despite the large reduction in emissions fluxes, burdens remained the same between time periods. This is likely the result of a combination of both increased CO from transported sources from the PNW combined with decreased local NO_X, that consequently reduces the source of OH and increases CO lifetime.

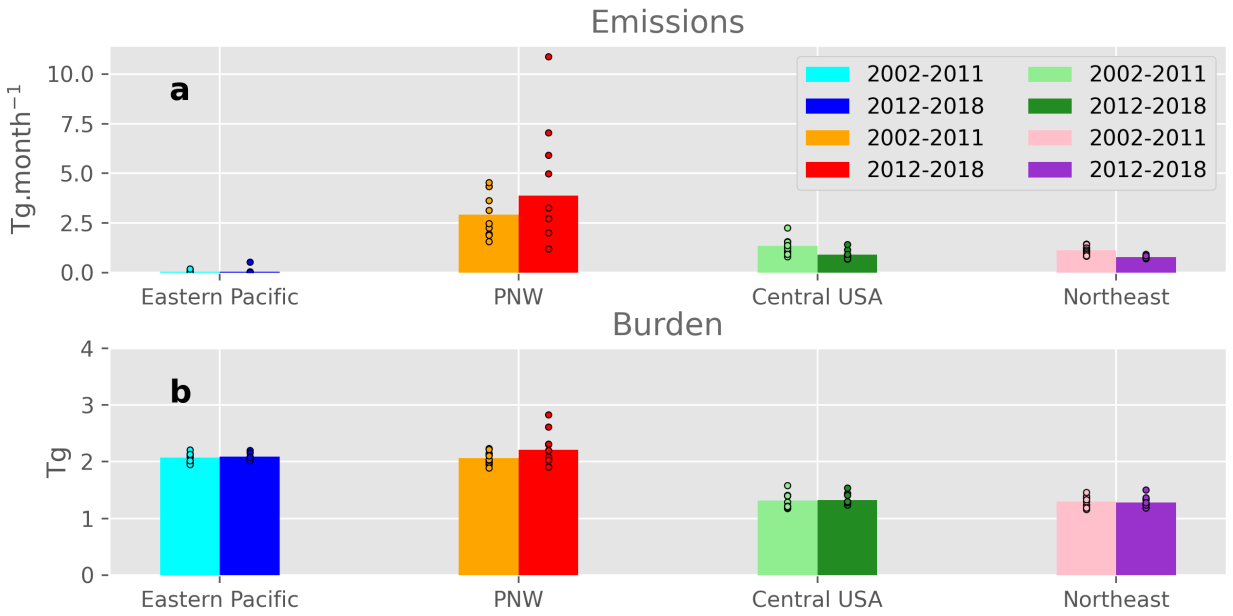

Supplementary Figure 11: Analysis of CAM-chem simulations. Regional average of the August sum for each period (2002 to 2011 and 2012 to 2018), (a) shows the CO emissions in terragrams per month (Tg month^{-1}), (b) the atmospheric burden in terragrams (Tg). Points represent the August sums for each region (n: Eastern Pacific = 408, PNW = 408, Central USA = 195, Eastern USA = 204).

In order to examine the potential extent of PNW wildfire emissions impact on North American air quality, we examine 2018 PNW wildfire season using a sensitivity simulation experiment. We perform full-chemistry simulations using CESM2.1/CAM-chem[10] with a model spin-up for the first 6 months of 2018. Horizontal resolution was $0.92° \times 1.25°$, with 56 levels of vertical resolution. Simulations were run with specified dynamics using MERRA2 nudged at 1%. Biogenic emissions were from Model of Emissions of Gases and Aerosols from Nature (MEGAN) coupled to CLM[11], anthropogenic emissions from CAMS-GLOB-ANT v3.1[12], and fire emissions from QFED[4].

Two simulations were carried out, one control simulation including all emissions and the second without PNW wildfire emissions from July 2018 onwards. In the second simulation, we masked fire emissions within a region that covered a large portion of the PNW extending from 32.5° N to 60° N, and 132.5° W to 109° W. Within this PNW region, all fire emissions

were set to zero thus limiting the effects of wildfires in the PNW in the model. Subtracting the mean surface CO of the masked simulation from the control shows large enhancements in CO over the PNW when fire emissions are included (Supp. Fig. 12). These enhancements extend across large regions of North America.

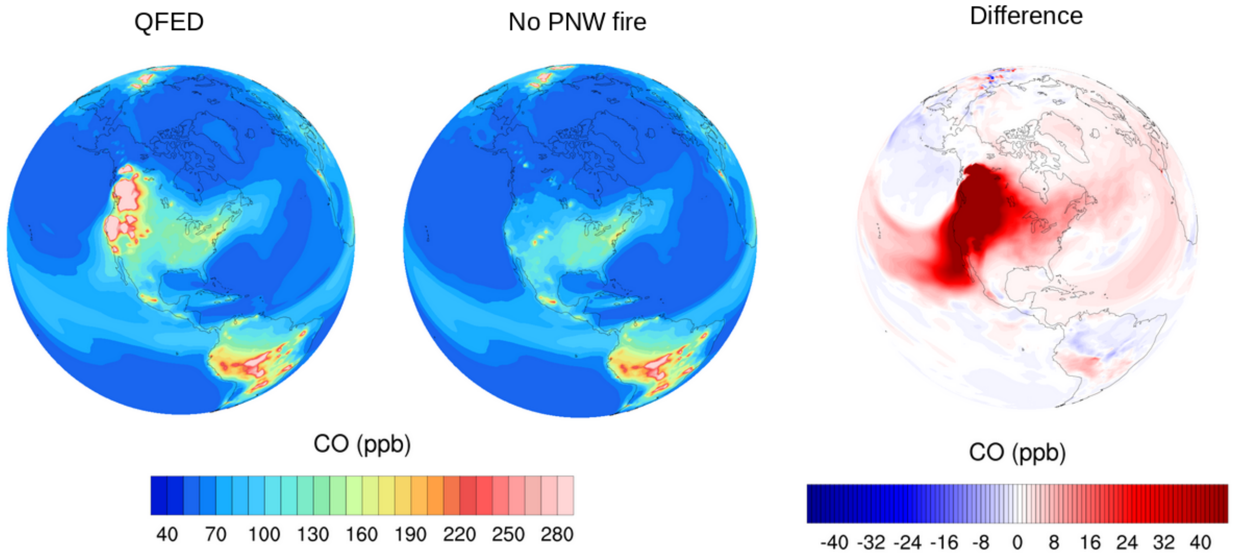

Supplementary Figure 12: Sensitivity study of PNW fire emissions using CAM-chem. Average surface CO for August 2018 from a CAM-chem simulation with QFEDv2.5 fire emissions (left) and without QFEDv2.5 fire emissions in the Pacific Northwest (center). The difference between simulations is shown on the right.

Using a full chemistry simulation allows us to examine the effect on other atmospheric species that are related to air quality. Equivalent differences between the masked simulation and control for ozone (O_3) and fine particulate matter ($PM_{2.5}$) show similar spatial extent of increased concentrations when including PNW wildfire emissions (Supp. Fig. 13). These simulations show that in 2018, and potentially other years with large wildfires, the PNW wildfires can have substantial impact on air quality locally, regionally and far-downwind, showing impact across large regions of the North America landmass. There are also increases in outflow pollution over the Pacific Ocean for August 2018 when including fire emissions.

In summary, these model simulations are a first look at the impact of PNW wildfire on local and downwind regions. A more complete modeling study would consider:

- Including tagged tracers to quantify the impact of each source region to the receptor regions. This will help quantify the PNW wildfire impact on the change in burden and CO lifetime in the Northeast and Central USA.

- The CMIP6 emissions do not accurately reflect the decrease in East Asian CO, so we do not see the expected decrease in East Pacific burden in Supplementary Figure 11.

Improved anthropogenic emissions from an inventory such as CAMS-GLOB-ANT v3.1 emissions could improve these results.

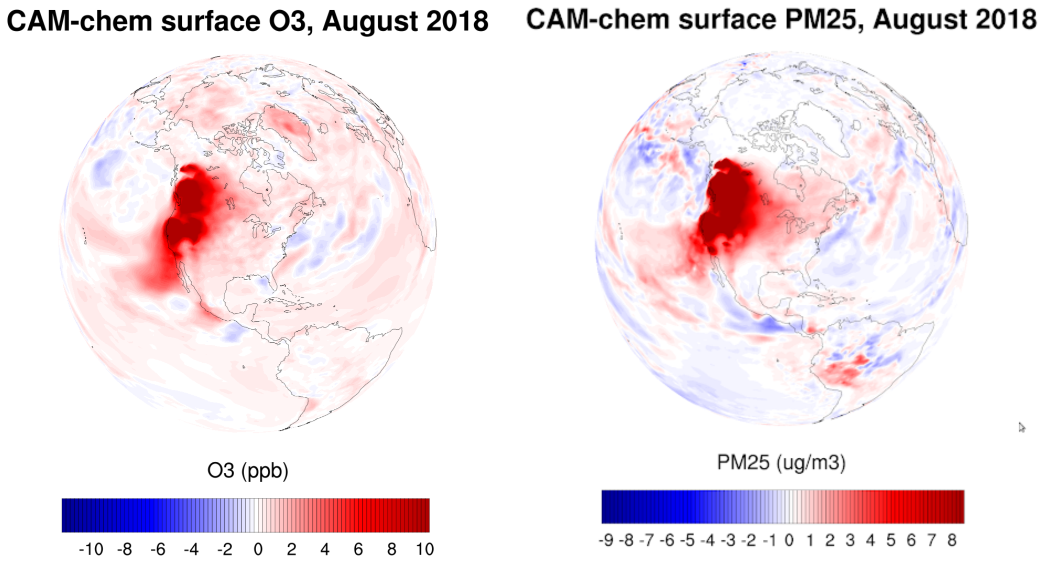

Supplementary Figure 13: Modeled PNW fire impact on surface atmospheric composition. Differences between including and not including PNW fire emissions in 2018 CAM-chem simulations for ozone (left) and $PM_{2.5}$ (right).

3 Supplementary Information Section 2: Potential health relationships

MOPITT shows seasonal pattern changes in surface CO (Fig. 3) and Supplementary Section 1 identified the potential impact of PNW wildfires on air quality relevant species at the surface for large regions of North America. Changes in air quality suggests that health responses may also be changing. Wildfire smoke exposure has been linked to cardiovascular and respiratory morbidity responses[13, 14, 15]. Although the mortality increases from short-term exposure to ambient $PM_{2.5}$ are well established[16], studies specific to short-term wildfire smoke exposure are limited. There is some evidence of cardiovascular mortality[17] and respiratory mortality[18]. As such, we briefly analyzed seasonal patterns for respiratory health in Colorado, a state in the Central USA region, to understand potential respiratory health responses to transported wildfire pollution in the late summer. We note that this is not the examination framework for an epidemiological study, where short-term effects need to be studied on daily basis using a time-series approach. By contrast, our focus is on if the special seasonal cycle we find in wildfire emissions may have potential indication on health responses.

We use monthly mortality data from the Vital Statistics Program, Colorado Department of Public Health & Environment. Data are separated into seasonal cycles for 2002-2011 (period 1) and 2012-2018 (period 2). Mortality annual cycle related to asthma and other chronic lower respiratory conditions (bronchitis, emphysema, COPD, bronchiolectasis) is shown in Supplementary Figure 14. We find other chronic respiratory conditions show a significant increase in August mortality for period 2 compared to period 1 for a Welch's independent two-tailed t-test at the $\alpha = .05$ level (t=-5.4, p=1.6×10^{-4}, df=12.8, mean difference(effect size)=6.4, 95% C.I.=3.8;9.0). Asthma mortality shows small decreases in mortality for the first half of the year for period 2 compared to period 1, while for the second half of the year there appears to be no difference between time periods.

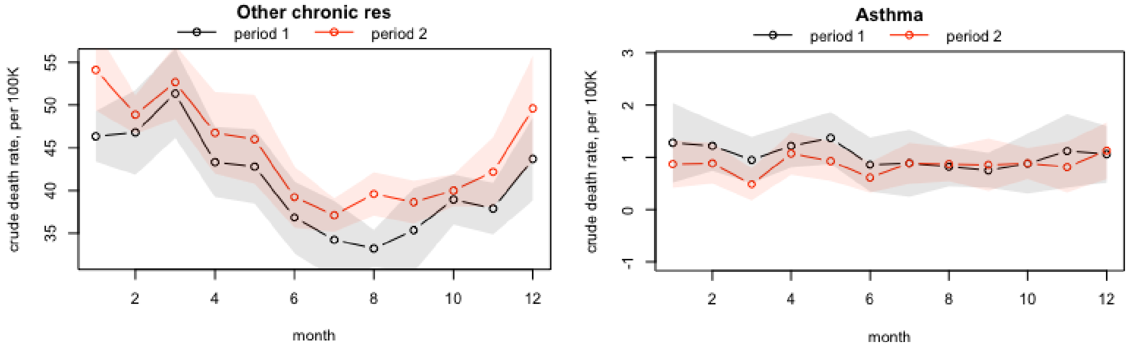

Supplementary Figure 14: Analysis of monthly mortality. Seasonal cycles of monthly population weighted other chronic respiratory mortality (left) and asthma mortality (right) in Colorado for time period 1 (black, 2002-2011) and time period 2 (red, 2012-2018). Shaded areas denote standard deviation.

We additionally plot the surface layer seasonal cycles of MOPITT CO in Colorado (37° to 41° N, 102° to 109° W), without detrending (Supp. Fig. 15, Supp. Table 1). The resulting pattern in CO has similarities with mortality seasonal cycle plots from both asthma and other chronic respiratory conditions. The first half of the CO annual cycle shows significant decreases in 2012-2018 relative to 2002-2011, while the second half of the annual cycle shows no significant changes between time periods (Supp. Table 1). Thus the pattern of change in Colorado surface CO shows a similar pattern to asthma mortality, with reductions in the early part of the year for 2012-2018 and no change for the second half of the year. Additionally, the emerging peak in August CO in the later period, coincides with the increased August peak in mortality for other chronic respiratory conditions for this same period. These pattern similarities between mortality and atmospheric composition suggest that there may be a relationship between changes in transported wildfire pollution and health. However, a full epidemiological study with detailed time series examination is required to accurately assign a causality relationship. Additionally, based on previous literature, hospital admissions and emergency room visits may show more significant response to wildfire pollution than mortality[14], and is the subject of future analysis.

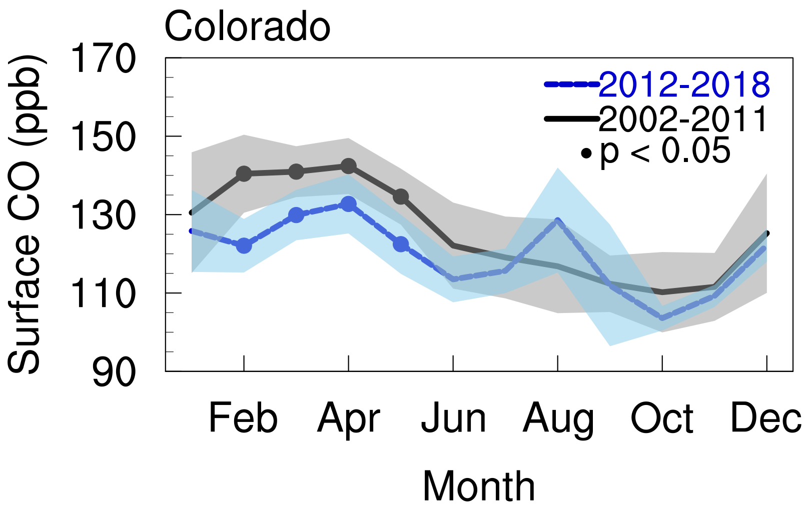

Supplementary Figure 15: Surface CO seasonality in Colorado. Seasonal cycle in MOPITT surface CO 2002-2011 and 2012-2018 (not detrended). Shaded area shows standard deviation and filled circles represent data means that are significantly different between the two time periods at the $\alpha = .05$ level according to a two-tailed t-test.

Supplementary Table 1: Comparison statistics from an independent, two-tailed t-test between 2002 to 2011 and 2012-2018, for Colorado surface CO (not detrended).

	P	t	df	d	Mean difference	95% CI
January	.498	0.694	15	0.354	4.663	-7.460; 6.785
February	.001	4.217	15	2.152	18.361	10.490; 26.232
March	.003	3.476	15	1.715	11.072	4.923; 17.220
April	.017	2.687	15	1.317	9.669	2.639; 16.698
May	.004	3.369	15	1.651	12.136	5.095; 19.176
June	.079	1.886	15	0.980	8.613	0.659; 16.568
July	.446	0.782	15	0.406	3.411	-4.204; 11.026
August	.084	-1.859	14	0.930	-11.794	-24.247; 0.658
September	.940	0.076	15	0.035	0.425	-11.734; 12.584
October	.121	1.642	15	0.873	6.618	-0.055; 13.292
November	.510	0.675	15	0.358	2.308	-3.373; 7.989
December	.615	0.514	15	0.274	3.058	-6.742; 12.858

Legend: P = P value, t = t statistic, df = degrees of freedom, d = Cohen's measure of sample effect size for comparing two sample means, Mean difference = 2002 to 2011 - 2012 to 2018, 95% CI = Confidence interval for 95%

Supplementary References

[1] Giglio, L., Boschetti, L., Roy, D. P., Humber, M. L. & Justice, C. O. The collection 6 modis burned area mapping algorithm and product. *Remote Sensing of Environment* **217**, 72–85 (2018). URL `https://www.sciencedirect.com/science/article/pii/S0034425718303705`.

[2] Giglio, L., Schroeder, W. & Justice, C. O. The collection 6 modis active fire detection algorithm and fire products. *Remote Sensing of Environment* **178**, 31–41 (2016). URL `https://www.sciencedirect.com/science/article/pii/S0034425716300827`.

[3] Wiedinmyer, C. *et al.* The Fire INventory from NCAR (FINN): a high resolution global model to estimate the emissions from open burning. *Geoscientific Model Development* **4**, 625–641 (2011). URL `https://www.geosci-model-dev.net/4/625/2011/`.

[4] Darmenov, A. & da Silva, A. The quick fire emissions dataset (QFED) – Documentation of versions 2.1, 2.2 and 2.4. NASA//TM-2015-104606 (2015). URL `https://gmao.gsfc.nasa.gov/pubs/docs/Darmenov796.pdf`.

[5] Wooster, M. J., Roberts, G., Perry, G. L. W. & Kaufman, Y. J. Retrieval of biomass combustion rates and totals from fire radiative power observations: FRP derivation and calibration relationships between biomass consumption and fire radiative energy release. *Journal of Geophysical Research: Atmospheres* **110** (2005). URL `https://agupubs.onlinelibrary.wiley.com/doi/abs/10.1029/2005JD006318`.

[6] van der Werf, G. R. *et al.* Global fire emissions estimates during 1997–2016. *Earth System Science Data* **9**, 697–720 (2017). URL `https://essd.copernicus.org/articles/9/697/2017/`.

[7] Randerson, J. T., Chen, Y., van der Werf, G. R., Rogers, B. M. & Morton, D. C. Global burned area and biomass burning emissions from small fires. *Journal of Geophysical Research: Biogeosciences* **117** (2012). URL `https://agupubs.onlinelibrary.wiley.com/doi/abs/10.1029/2012JG002128`.

[8] Zheng, B. *et al.* Global atmospheric carbon monoxide budget 2000–2017 inferred from multi-species atmospheric inversions. *Earth System Science Data* **11**, 1411–1436 (2019). URL `https://essd.copernicus.org/articles/11/1411/2019/`.

[9] Hoesly, R. M. *et al.* Historical (1750–2014) anthropogenic emissions of reactive gases and aerosols from the Community Emissions Data System (CEDS). *Geoscientific Model Development* **11**, 369–408 (2018). URL `https://gmd.copernicus.org/articles/11/369/2018/`.

[10] Emmons, L. K. *et al.* The chemistry mechanism in the Community Earth System Model version 2 (CESM2). *Journal of Advances in Modeling Earth Systems*

12, e2019MS001882 (2020). URL https://agupubs.onlinelibrary.wiley.com/doi/abs/10.1029/2019MS001882.

[11] Guenther, A. B. *et al.* The Model of Emissions of Gases and Aerosols from Nature version 2.1 (MEGAN2.1): an extended and updated framework for modeling biogenic emissions. *Geoscientific Model Development* **5**, 1471–1492 (2012). URL https://gmd.copernicus.org/articles/5/1471/2012/.

[12] Granier, C. *et al.* The Copernicus Atmosphere Monitoring Service global and regional emissions (April 2019 version). *Copernicus Atmosphere Monitoring Service (CAMS) report* (2019). URL https://doi.org/10.24380/d0bn-kx16.

[13] Reid, C. E. *et al.* Critical review of health impacts of wildfire smoke exposure. *Environmental Health Perspectives* **124**, 1334–1343 (2016). URL https://ehp.niehs.nih.gov/doi/abs/10.1289/ehp.1409277.

[14] Gan, R. W. *et al.* The association between wildfire smoke exposure and asthma-specific medical care utilization in Oregon during the 2013 wildfire season. *J Expo Sci Environ Epidemiol* **30**, 618–628 (2020). URL https://doi.org/10.1038/s41370-020-0210-x.

[15] Gan, R. W. *et al.* Comparison of wildfire smoke estimation methods and associations with cardiopulmonary-related hospital admissions. *GeoHealth* **1**, 122–136 (2017). URL https://agupubs.onlinelibrary.wiley.com/doi/abs/10.1002/2017GH000073. https://agupubs.onlinelibrary.wiley.com/doi/pdf/10.1002/2017GH000073.

[16] EPA. Integrated Science Assessment for Particulate Matter. *US Environmental Protection Agency* (2020). URL https://www.epa.gov/isa/integrated-science-assessment-isa-particulate-matter.

[17] Kollanus, V., Tiittanen, P., Niemi, J. V. & Lanki, T. Effects of long-range transported air pollution from vegetation fires on daily mortality and hospital admissions in the helsinki metropolitan area, finland. *Environmental Research* **151**, 351 – 358 (2016). URL http://www.sciencedirect.com/science/article/pii/S001393511630353X.

[18] Faustini, A. *et al.* Short-term effects of particulate matter on mortality during forest fires in southern europe: results of the med-particles project. *Occupational and Environmental Medicine* **72**, 323–329 (2015). URL https://oem.bmj.com/content/72/5/323.
