# Supplementary material for: New seasonal pattern of pollution emerges from changing North American wildfires
Source: Nat Commun. 2022 Apr 19;13:2043. doi: 10.1038/s41467-022-29623-8 (PMC9018720; doi:10.1038/s41467-022-29623-8)
Supplement: Supplementary file 3 — Reporting Summary [file 41467_2022_29623_MOESM3_ESM.pdf]

nature portfolio

Corresponding author(s): Rebecca R. Buchholz

Last updated by author(s): Feb 15, 2022

Reporting Summary

Nature Portfolio wishes to improve the reproducibility of the work that we publish. This form provides structure for consistency and transparency in reporting. For further information on Nature Portfolio policies, see our Editorial Policies and the Editorial Policy Checklist.

Statistics

For all statistical analyses, confirm that the following items are present in the figure legend, table legend, main text, or Methods section.

n/a	Confirmed	
☐	☒	The exact sample size (n) for each experimental group/condition, given as a discrete number and unit of measurement
☐	☒	A statement on whether measurements were taken from distinct samples or whether the same sample was measured repeatedly
☐	☒	The statistical test(s) used AND whether they are one- or two-sided<br>*Only common tests should be described solely by name; describe more complex techniques in the Methods section.*
☒	☐	A description of all covariates tested
☐	☒	A description of any assumptions or corrections, such as tests of normality and adjustment for multiple comparisons
☐	☒	A full description of the statistical parameters including central tendency (e.g. means) or other basic estimates (e.g. regression coefficient) AND variation (e.g. standard deviation) or associated estimates of uncertainty (e.g. confidence intervals)
☐	☒	For null hypothesis testing, the test statistic (e.g. F, t, r) with confidence intervals, effect sizes, degrees of freedom and P value noted<br>*Give P values as exact values whenever suitable.*
☒	☐	For Bayesian analysis, information on the choice of priors and Markov chain Monte Carlo settings
☒	☐	For hierarchical and complex designs, identification of the appropriate level for tests and full reporting of outcomes
☐	☒	Estimates of effect sizes (e.g. Cohen's d, Pearson's r), indicating how they were calculated

Our web collection on statistics for biologists contains articles on many of the points above.

Software and code

Policy information about availability of computer code

Data collection	N/A - we used publicly available data.
Data analysis	Data analysis was completed using a combination of Excel, IDL v8.7.3, Matlab R2015b and NCL v6.6.2 code. Code used to analyze MOPITT and MODIS trends, as well as seasonal cycles is available via GitHub (https://github.com/rrbuchholz/fire_atmosphere_analysis_2022, doi: 10.5281/zenodo.6049601).

For manuscripts utilizing custom algorithms or software that are central to the research but not yet described in published literature, software must be made available to editors and reviewers. We strongly encourage code deposition in a community repository (e.g. GitHub). See the Nature Portfolio guidelines for submitting code & software for further information.

Data

Policy information about availability of data

All manuscripts must include a data availability statement. This statement should provide the following information, where applicable:

- Accession codes, unique identifiers, or web links for publicly available datasets
- A description of any restrictions on data availability
- For clinical datasets or third party data, please ensure that the statement adheres to our policy

Data used in this analysis is publicly available. MOPITT CO is available at https://dx.doi.org/10.5067/TERRA/MOPITT/MOP02J_L2.008. MODIS AOD is available from https://dx.doi.org/10.5067/MODIS/MOD08_M3.061. Emissions inventories are available from their respective repositories: QFED2.5 -- https://portal.nccs.nasa.gov/datashare/iesa/aerosol/emissions/QFED/v2.5r1/0.25/QFED/; FINN1.5 -- http://bai.acom.ucar.edu/Data/fire/; GFED4.1s -- https://globalfiredata.org/pages/data/; CAMS-GLOB-ANT v3.1 -- accessed from https://eccad3.sedoo.fr/, archive available from https://ads.atmosphere.copernicus.eu/cdsapp#!/dataset/cams-global-emission-inventories; Zheng Reanalysis -- https://doi.org/10.6084/m9.figshare.c.4454453.v1. CAM-chem output used to analyze 2002-2018 is available from the

NCAR Research Data Archive at https://rda.ucar.edu/datasets/ds313.7. The MODIS burned area and fire count Climate Modeling Grid products were accessed via sftp from fuoco.geog.umd.edu. Source data used in main manuscript Figures 2 to 5 are provided with this paper. Mortality data were accessed via Wide-ranging OnLine Data for Epidemiologic Research (WONDER) from the National Vital Statistics System at the National Center for Health Statistics, Centers for Disease Control, available at https://wonder.cdc.gov/Deaths-by-Underlying-Cause.html.

Field-specific reporting

Please select the one below that is the best fit for your research. If you are not sure, read the appropriate sections before making your selection.

☐ Life sciences ☐ Behavioural & social sciences ☒ Ecological, evolutionary & environmental sciences

For a reference copy of the document with all sections, see nature.com/documents/nr-reporting-summary-flat.pdf

Ecological, evolutionary & environmental sciences study design

All studies must disclose on these points even when the disclosure is negative.

Study description	The study uses satellite-based measurements of atmospheric composition, fire emission inventories, global modeling and Colorado health data to investigate the impact of increasing fires in the Pacific Northwest (PNW) on air pollution 2002-2018 and the potential impacts on human health.
Research sample	Atmospheric composition is measured from satellite-based instruments. Carbon monoxide (CO) and Aerosol Optical Depth (AOD) are chosen due to their ability to demonstrate an atmospheric response to fire. Although measurements are global, we focus on North America because of the recent extreme fire seasons. CO is also investigated using four fire emission inventories and two anthropogenic emissions inventories to support our conclusion that fire is the main driver of the observed atmospheric response. The case-study of human respiratory mortality in Colorado is an example of possible human health response to fires, to show the implications of increasing fires.<br><br>MOPITT CO is available at https://dx.doi.org/10.5067/TERRA/MOPITT/MOP02J_L2.008. MODIS AOD is available from https://dx.doi.org/10.5067/MODIS/MOD08_M3.061. Fire emissions inventories are available from their respective repositories: QFED2.5 -- https://portal.nccs.nasa.gov/datashare/iesa/aerosol/emissions/QFED/v2.5r1/0.25/QFED/; FINN1.5 -- http://bai.acom.ucar.edu/Data/fire/; GFED4.1s -- https://globalfiredata.org/pages/data/; CAMS-GLOB-ANT v3.1 -- accessed from https://eccad3.sedoo.fr/, archive available from https://ads.atmosphere.copernicus.eu/cdsapp#!/dataset/cams-global-emission-inventories; Zheng Reanalysis -- https://doi.org/10.6084/m9.figshare.c.4454453.v1. CAM-chem output used to analyze 2002-2018 is available from the NCAR Research Data Archive at https://rda.ucar.edu/datasets/ds313.7. The MODIS burned area and fire count Climate Modeling Grid products were accessed via sftp from fuoco.geog.umd.edu. Mortality data were accessed via Wide-ranging OnLine Data for Epidemiologic Research (WONDER) from the National Vital Statistics System at the National Center for Health Statistics, Centers for Disease Control, available at https://wonder.cdc.gov/Deaths-by-Underlying-Cause.html.
Sampling strategy	Measurements Of Pollution In The Troposphere (MOPITT) is a nadir-viewing gas correlation radiometer measuring in the thermal infrared (TIR) near 2140 wavenumbers and at 4275 wavenumbers in the near infrared NIR at an overpass of 10:30 am and pm local time. Global coverage occurs about every three days with a ground resolution of ~22 square km at nadir. Optimal estimation on gas cell absorption retrieves CO profiles of dry air volume mixing ratio (VMR) on 10 vertical layers, which are integrated to column amounts. The Moderate Resolution Imaging Spectroradiometer (MODIS) is a passive imaging radiometer, measuring reflected solar and thermal radiation in 36 bands, with global coverage in ~1 day at spatial resolution between 250m and 1km at nadir. We use AOD at 550 nm from the merged Dark Target Dark Blue (DTDB) product. We monthly average the atmospheric composition observations over large regions in North America: the Pacific Northwest (PNW, 38 to 57 deg N, 127 to 110 deg W), Central USA (35 to 49 deg N, 110 to 95 deg W), and the Northeast (41 to 53 deg N, 95 to 74 deg W). Monthly regional averaged data was used for atmospheric composition data for sufficient statistics, while retaining the ability to demonstrate seasonal patterns. For the CO observations, which are regional averages of all retrievals within a month, the mean sample size is PNW: 1110, Central USA: 2469, and Northeast: 1341. For the AOD data, which are regional averages of monthly average data, the mean sample size is: PNW: 240, Central USA: 190 and Northeast: 175. Mortality data is provided as the monthly annualized death rate per 100,000 population for all of the State of Colorado, USA. Colorado population ranged between 4.5 million in 2002 and 5.7 million in 2018. We averaged the monthly health data into two time periods 2002-2011 and 2012-2018.
Data collection	We used publicly available data. NASA satellite data sets are self-described in their doi url links. We selected MOPITT CO because it is the longest running measurements of CO from space. MODIS AOD was used because it is measured from the same satellite platform, Terra. Data is publicly provided as hdf files. Mortality data were accessed via Wide-ranging OnLine Data for Epidemiologic Research and are collected and provided by the Colorado Department of Public Health and Environment (CDPHE).
Timing and spatial scale	MOPITT CO: 2002-2018, 22km ground resolution, global coverage.<br>MODIS: 2002-2018, 250 m to 1km spatial resolution, global coverage.<br>Regions of interest are the Pacific Northwest (PNW, 38 to 57 deg N, 127 to 110 deg W), Central USA (35 to 49 deg N, 110 to 95 deg W), and the Northeast (41 to 53 deg N, 95 to 74 deg W).<br>Colorado Vital Statistics mortality data: the state of Colorado, monthly annualized death rate per 100,000 population, 2002-2018.
Data exclusions	MOPITT CO in August 2009 was missing due to instrument down-time.
Reproducibility	We tested different end points for the CO August trend analysis (2016, 2017 and 2018) and all end points showed consistent increasing trends in CO over the PNW.

Randomization	Randomization was not relevant - we split the data record into two based on the emergence of a secondary annual peak.
Blinding	Blinding was not relevant to our study because we are not introducing an intervention.

Did the study involve field work? ☐ Yes ☒ No

Reporting for specific materials, systems and methods

We require information from authors about some types of materials, experimental systems and methods used in many studies. Here, indicate whether each material, system or method listed is relevant to your study. If you are not sure if a list item applies to your research, read the appropriate section before selecting a response.

Materials & experimental systems

n/a	Involved in the study
☒	☐ Antibodies
☒	☐ Eukaryotic cell lines
☒	☐ Palaeontology and archaeology
☒	☐ Animals and other organisms
☒	☐ Human research participants
☒	☐ Clinical data
☒	☐ Dual use research of concern

Methods

n/a	Involved in the study
☒	☐ ChIP-seq
☒	☐ Flow cytometry
☒	☐ MRI-based neuroimaging
